# Investigation on Molecular Mechanism of Fibroblast Regulation and the Treatment of Recurrent Oral Ulcer by Shuizhongcao Granule-Containing Serum

**DOI:** 10.1155/2015/324091

**Published:** 2015-10-18

**Authors:** Zhang Bo, Qian Xiang, Ruan Shan-ming, Bei Wang, Deng De-hou, Xia Liang, Li Qing-lin, Tao Feng, Shen Min-he

**Affiliations:** ^1^Zhejiang Cancer Hospital, Hangzhou, Zhejiang 310022, China; ^2^Zhejiang Chinese Medical University, Hangzhou, Zhejiang 310053, China; ^3^The First Affiliated Hospital of Zhejiang Chinese Medical University, Hangzhou, Zhejiang 310006, China; ^4^Zhejiang Medical College, Hangzhou, Zhejiang 310053, China

## Abstract

The purpose is to study the intervention, proliferation, and differentiation on fibroblast by Shuizhongcao Granule during the treatment of ROU and investigate the action mechanism in inflammatory microenvironment. Proliferation of rat fibroblasts was detected using CCK8. Western blot was used to detect the effect of drug-containing serum on the expression of protein associated with NF-*κ*B and ERK pathway in rat fibroblasts. Expression of IL-10 and IL-12 was detected by PCR. Shuizhongcao Granule group successfully inhibited proliferation of rat fibroblast. Western blot results revealed that p65 and IKKB were significantly less expressed in Chinese medicine group, while pI*κ*B*α* and pIKK*αβ* expression were significantly increased. We have also found that in this group the expression of pAKT was evidently suppressed and expression of pERK significantly decreased. PCR results showed significantly decreased expression levels of IL-10 and 1IL-12b in Chinese medicine group, while the expression of IL-12a was increased. Our results suggest that Shuizhongcao Granule can suppress the proliferation of fibroblast and inhibit the activation of NF-*κ*B and thus suppress inflammatory reactions, possibly involving the inhibited expression of phosphorylated AKT, rather than the canonical pathway. Furthermore, it can inhibit ERK pathway and reduce IL-10 and IL-12b gene expression while enhancing IL-12a expression.

## 1. Introduction

Recurrent oral ulcer (ROU) is the most frequent oral mucosal disease with an incidence rate up to 25–30% [1]. The main type, minor ROU, accounts for 70–80% of all cases [2]. ROU is also one of the most common complications of chemotherapy. The current treatments mainly include antibiotic therapy [3], hormonal therapy [4], medicine mouthwash [5], and laser therapy [6], but none of them proves to be very effective. Finding a safe and effective medication to suppress inflammation is a hot and difficult research topic in this field. Suppression of NF-*κ*B pathway to reduce the incidence gives a novel resolution to treat ROU [7]. From the perspective of traditional Chinese medicine, ROU is a type of “aphthae,” which is usually considered to be associated with the phase of “fire.” A proven formula in our hospital, Shuizhongcao Decoction (composed of buffalo horn, urine sediment,* Callicarpa*, etc.), exhibited great efficacy in the clinical treatment through a mechanism of heat reduction, blood cooling, and detoxification [8]. This study evaluated the intervention effects of serum containing Shuizhongcao on rat fibroblasts and tested the optimal dosage and intervention time. We also detected the effect of Shuizhongcao Granule on ERK and NF-*κ*B pathways using Western blot and expression of IL-12 and IL-10 by RT-PCR. Therein, we evaluated the effect and the mechanism of Shuizhongcao Granule on the inflammatory microenvironment and the rat fibroblasts. The results are reported as follows.

## 2. Materials

### 2.1. Experimental Cells

Rat ear-tip fibroblasts were purchased from Shanghai SiDanSai Biotechnology, Ltd. (lot number 1702-100).

### 2.2. Experimental Animals

Sixty Sprague-Dawley (SD) rats in healthy and hygiene grade (weighted 180 ± 20 g, 6–8 weeks, half male and half female) were provided by Laboratory Animal Center of Zhejiang Chinese Medical University.

### 2.3. Chemicals and Medicines

5-FU was purchased from Zhejiang Chinese Medical Hospital, manufactured by Tianjin Jinyao Amino Acid, Ltd. (10 mL: 0.25 g, lot number 0909162, National Medicine Permit number H12020959). Chloral hydrate was purchased from Zhejiang Chinese Medical Hospital (specification 30 m: 3 g, 30 mL/bottle, Zhejiang Medicine Permit H20050357). NaOH crystal was purchased from Eastern China Medicine Ltd., manufactured by Hangzhou Xiaoshan Chemical Reagent Manufacturing (lot number 201003020). Shuizhongcao Granule (buffalo horn tablet 30 g, burned urine sediment 15 g, and* Callicarpa* leaf 15 g), 2.0 g crude drug per milliliter, was purchased from Chinese Medicine Pharmacy of Zhejiang Chinese Medical Hospital (buffalo horn, produced in Zhejiang, lot number 130112; burned urine sediment, produced in Anhui, lot number 121103;* Callicarpa* leaf, produced in Zhejiang, lot number 120922).

Preparation of medicine is as follows: buffalo horn tablets, burned urine sediment, and* Callicarpa* leaf (2 : 1 : 1 in weight) were soaked in pure water of 8–10 times in volume for 30 min. Buffalo horn tablets were boiled alone for 30 min and then added with burned urine sediment and* Callicarpa* leaf. After boiling with high heat, the medicines were then boiled with low heat for 30 min. For the second time of boiling, the herb residues were added with pure water of 1.5 times in volume and boiled for another 30 min. The decoctions from the two boiling medcine juice were then condensed to a concentration with containing crude drug 2.0 g/mL. The medicines were sterilized and packed for use.

### 2.4. Reagents

The reagents are trypsin (product number 1310S, Jinuo Biomedical Technology, Hangzhou, Zhejiang, China), fibroblast complete culture media (product number 0810-500, Si Dan Sai Biotechnology, Shanghai, China), CCK8 (catalog number CK04, Do jin do Molecular Technologies, Tokyo, Japan), NF-*κ*B signaling pathway kit (product number 9936S, Cell Signaling Technology, Santa Cruz, CA, USA), MAPK signaling pathway kit (product number 8922S, Cell Signaling Technology, Santa Cruz, CA, USA), SIRT1 antibody (product number 9475S, Cell Signaling Technology, Santa Cruz, CA, USA), Immobilon-P membrane (PVDF, Shanghai Bai wei Biotechnology, Shanghai, China), RIPA lysis buffer (RIPA, Beyotime Institute of Biotechnology, Hangzhou, Zhejiang, China), BCA standard protein (product number 10735108001, Roche, Danvers, MA, USA), phosphatase inhibitor (number P5726-1 mL, Sigma-Aldrich, WI, USA), protease inhibitor (product number WBP1265-1 mL, Sigma-Aldrich, WI, USA), 5x-loading-buffer (number WB11236-1 mL, Sigma-Aldrich, WI, USA), anti-beta-actin (Actb, 1 : 3000, Sigma-Aldrich, WI, USA), total RNA extraction kit (product number: 15596-026, Invitrogen, New York, USA), Trizol (product number 1226-210, Invitrogen, New York, USA), PCR reverse transcription kit (product number DRR037A, TaKaRa Bio Inc., Palo Alto, CA, USA), and SYBR Premix Ex TaqTM (product number RR420A, Palo Alto, CA, USA).

### 2.5. Laboratory Apparatus

5% CO_2_ incubator HEPA Class 100 was purchased from Thermo Fisher. Water bath thermostat oscylator was bought from Taicang Laboratory Instrument Manufacture. CO_2_ incubator (5410-220) was made by Precision Scientific. Laminar flow hood was made by Suzhou Antai Air Technology Co., Ltd. (BCM-1000A). Counter top centrifuge was purchased from Beijing Jingli Centrifuge Co., Ltd. (LDZ 5-2). Electric Thermostat water bath (DK-450 B type) was purchased from Shanghai Senxin Experimental Instrument Co., Ltd. Liquid nitrogen tank was MVE CRYOSYSTEM750. Microplate reader was made by Thermo Fisher (3001-1249). UV spectrophotometer was made by Eppendorf. PCR gene amplifier was made by Bio-Rad Laboratories, Inc. (US). iQTM5 real-time quantitative PCR was made by Bio-Rad Laboratories, Inc. (US).

### 2.6. Preparation of Reagents

Cell lysis buffer (0.25% pancreatic enzyme +EDTA) was purchased freshly and stored in freezer at −20°C. PBS buffer (pH 7.2–7.4) was purchased freshly and stored in refrigerator at 4°C. 30% acrylamide/bis solution (propylene thalidomide 0.8 g and N-methylene bisacrylamide thalidomide 29.2 g in dd-water to a final volume of 100 mL) was made in a fume hood, stored at room temperature, and protected from light. 10% SDS (sodium dodecyl sulfate) was made by dissolving 10 g SDS in dd-water to a final volume of 100 mL. 10% ammonium persulfate (0.1 g ammonium persulfate in 1.0 mL dd-water) was made freshly and stored at 4°C less than a week. 15 mL 10% separating gel was prepared in the fume hood with 6 mL dd-water, 5 mL 30% acrylamide/bis solution, 3.75 mL pH 8.8 Tris-HCL, 150 *μ*L 10% SDS, 150 *μ*L 10% ammonium persulfate, and 7.5 *μ*L TEMED. The gel was mixed and loaded immediately after TEMED was added. 5 mL 5% stacking gel was prepared in the fume hood with 3.75 mL dd-water, 0.67 mL 30% acrylamide/bis solution, 0.62 mL pH 6.8 Tris-HCL, 50 *μ*L 10% SDS, 50 *μ*L 10% ammonium persulfate, and 5 *μ*L TEMED. The gel was mixed and loaded immediately after TEMED was added. 1x electrophoresis buffer was made by diluting 100 mL 10x electrophoresis buffer in dd-water to a final volume of 1000 mL. 1x transfer buffer was made by mixing 3.03 g Tris, 14.4 g glycine, and 200 mL methanol in dd-water to a final volume of 1000 mL. 10x TBS was made by dissolving 24.2 g Tris and 80 g NaCl in dd-water to a final volume of 800 mL, adjusting pH to 7.5 using appropriate amount of HCl, and bringing up to 1000 mL dd-water. 1x TBST was prepared by mixing 10x TBS 100 mL and Tween 20 1 mL in dd-water to a final volume of 1000 mL. Blocking buffer was made by dissolving 5 g fat-free milk powder or bovine serum albumin (BSA) in 100 mL 1x TBST.

## 3. Methods

### 3.1. Experimental Animal and Group Assignment

Sixty healthy rats (180 ± 20 g, 6–8 weeks, half male and half female) were fed for an acclimated period of a week and then randomized into three groups: blank control group, saline group, and Shuizhongcao treatment group.

### 3.2. Establishment of Experimental Animal Model

The rats were intraperitoneally administrated with 5-fluorouracil (5-Fu) with a dose of 5 mg/100 g for chemotherapy. On the fourth day after chemotherapy, rats were anesthetized with 10% chloral hydrate intraperitoneal injection (0.3 mL/100 mL). A piece of NaOH of appropriate size (1.5 mm × 1.5 mm) was placed by a straight head tweezer on the rat's right side cheek mucosa for 5–10 seconds until the naked appearance of redness and ulcer. Afterward all three groups were fed with normal diet. From the second day after surgery, saline group and Chinese medicine group were intragastrically administrated with standard saline and Shuizhongcao (1 mL/100 g) twice a day, respectively, for a total of 10 days. No additional treatment was applied to the blank control group.

### 3.3. Preparation of Rat Serum

At day 11 after surgery, the rats were anesthetized with 10% chloral hydrate intraperitoneal injection (0.3 mL/100 mL) and euthanized by abdominal aorta exsanguination. The peripheral blood obtained from rats was pipetted into a centrifuge tube containing 10% EDTA and pancreatic enzymes. After 10 min centrifugation at 200 g, the supernatant, which was the serum, was collected and stored in an ultralow freezer at −80°C.

### 3.4. Culture of Rat Fibroblast

Rat fibroblasts were cultured in fibroblast complete media and passaged when they reached confluence. After being subcultured for 6-7 passages, the cells reached the log phase of growth and were ready for experiment.

### 3.5. Detection of Drug-Containing Serum on Fibroblast by CCK8 Assay

The fibroblasts in log growth phase were counted and seeded in three 96-well plates, labeled with 12 h, 24 h, and 36 h, respectively. Each plate was divided into ten groups and each group contained 7 subgroups: 5%, 10%, and 15% Chinese medicine group and 5%, 10%, and 15% saline group and blank group. After 12 h, 24 h, and 36 h incubation, the corresponding 96-well plates were taken out, and CCK8 solution was added to each well with 10 *μ*L and continued incubation for 3h. The absorbance was detected and growth curve was plotted.

### 3.6. NF-*κ*B and ERK Pathway Protein Expression Detected by Western Blot

Cells from Chinese medicine group, saline group, and blank group were collected, washed in PBS solution, and lysed with lysis buffer. Total protein was extracted and quantified using BCA assay. 40 *μ*L protein sample was loaded in each well for SDS-PAGE. The protein was transferred from polyacrylamide gel to PVDF membrane by electrotransfer. The PVDF membrane with transferred protein was blocked with TBST containing 5% BSA (bovine serum albumin) overnight. The membrane was incubated with primary antibody (1 : 1000) for 2 h at 37°C and washed with TBS 3 times, 10 min each time. The membrane was then incubated with secondary antibody (1 : 5000) for 2 h at 37°C and washed with TBS 3 times, 10 min each time. The membrane was developed using ECL solutions and imaged.

### 3.7. Expression of IL-10, IL-12a, and IL-12b in Rat Fibroblasts in Each Group Detected by Real-Time Quantitative PCR

Total mRNA of cells from each group was extracted at 48 h after serum treatment using absorption column centrifugal method. After concentration, purity, and integrity assessment, mRNA was reversely transcribed into cDNA following the instruction of reverse transcription kit. Levels of IL-10, IL-12a, and IL-12b were detected by fluorescence-based quantitative RT-PCR. Primers were designed using software Primer Premier 6.0 and Beacon Designer and synthesized by Shenggong Bioengineering Technology Co., Ltd. (Shanghai). Primer sequences are listed in Table 1.

Reaction conditions were as follows: reverse transcription, 37°C for 15 min and 85°C for 5 sec; PCR reaction was as follows: 95°C, for 5 sec and 60°C for 30 sec (annealing), 40 cycles (fluorescence collection); melting curve analysis was 50°C to 95°C.

### 3.8. Statistical Analysis

Quantitative data were expressed as mean ± standard deviation ($\bar{X}\pm S$). SPSS 17.0 statistical software was used for statistical and variance analysis.

## 4. Results

### 4.1. Rat Model Establishment

12 h after rat model establishment, buccal mucosal vasodilatation, increased capillary network, and tissue edema were observed. At 24 h, edema became serious. The mucosal epithelial layer slightly exfoliated and the surface was covered with exudates. At 48 h, the oral ulcer formed, and the mucosa turned red extensively, which was covered by large pieces of pseudomembranes, appearing yellowish white in color and easily exfoliated. A specialist in pathology confirmed the diagnosis and the success of model establishment.

### 4.2. Culture of Rat Fibroblasts

The rat fibroblast culture grew well after subculturing.

### 4.3. CCK8 Results of Fibroblast Intervention by Control Serum and Drug-Containing Serum

**Figure 1:**
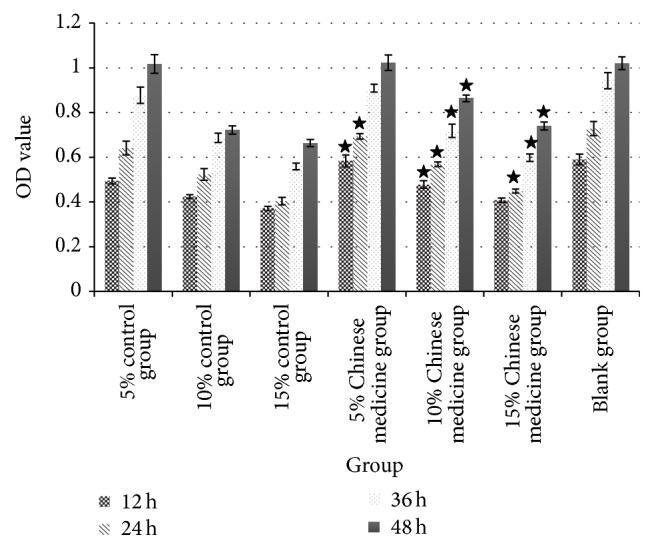
Cell growth and time relationship following serum intervention in control group and Chinese medicine group. Note: ^★^ *P* < 0.05 between Chinese medicine group and control group at the same time point.

As shown in Table 2 and Figure 1, serum in both control group and Chinese medicine group was able to suppress rat fibroblast proliferation, significantly different from that in blank group (*P* < 0.05). The suppressive effect was positively, but not proportionally, correlated with dosage and treatment time. The serum containing Chinese medicine reduced the suppressive effect of inflammation on rat fibroblasts. The difference between each group was statistically significant (*P* < 0.05).

### 4.4. Western Blot Results following Serum Intervention of Rat Fibroblasts

#### 4.4.1. Expression of Protein Associated with NF-*κ*B Pathway

**Figure 2:**
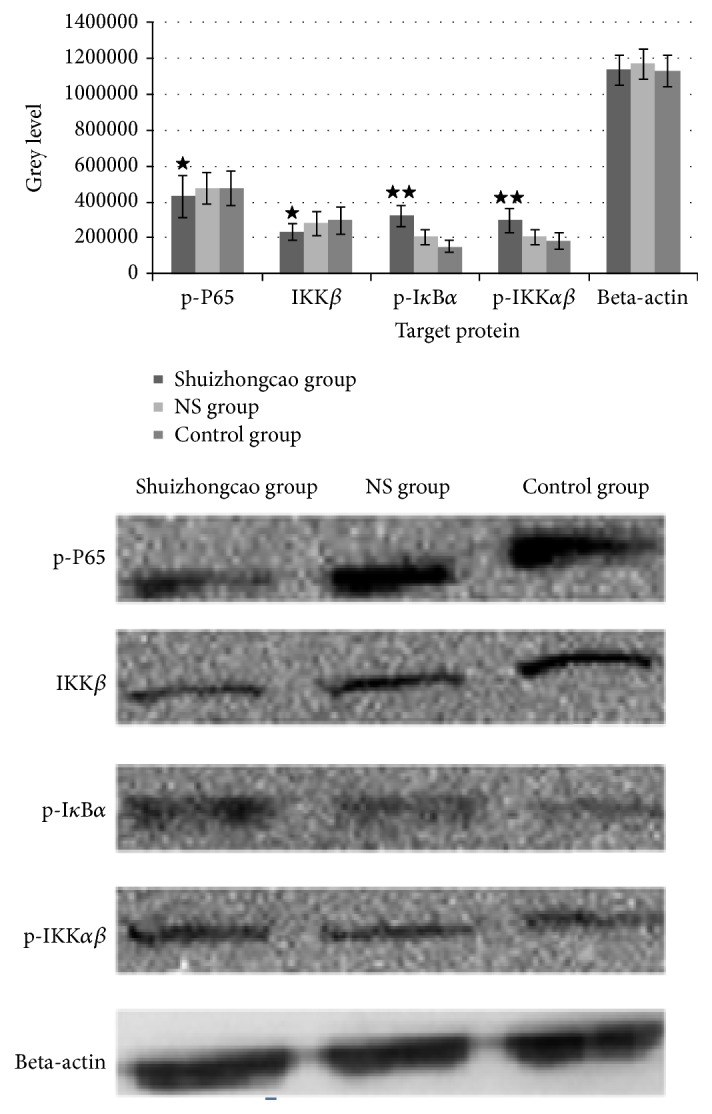
Protein expression of NF-*κ*B pathway.

The results showed that serum containing Shuizhongcao Granule significantly downregulated the expression of phosphorylated P65, demonstrating its ability to suppress the activation and relevant protein expression of NF-*κ*B pathway. In the meantime, Shuizhongcao increased phosphorylated I*κ*B*α* and phosphorylated IKK*αβ* expression, facilitating the activation of canonical NF-*κ*B pathway. The differences between each parameter from Shuizhongcao group (except for internal standard) and that of two other groups were statistically significant (*P* < 0.05). Differences between NS group (NS group is short for “normal saline group”) and control group were not significant. See Figure 2.

#### 4.4.2. Expression of Phosphorylated ERK1/2

**Figure 3:**
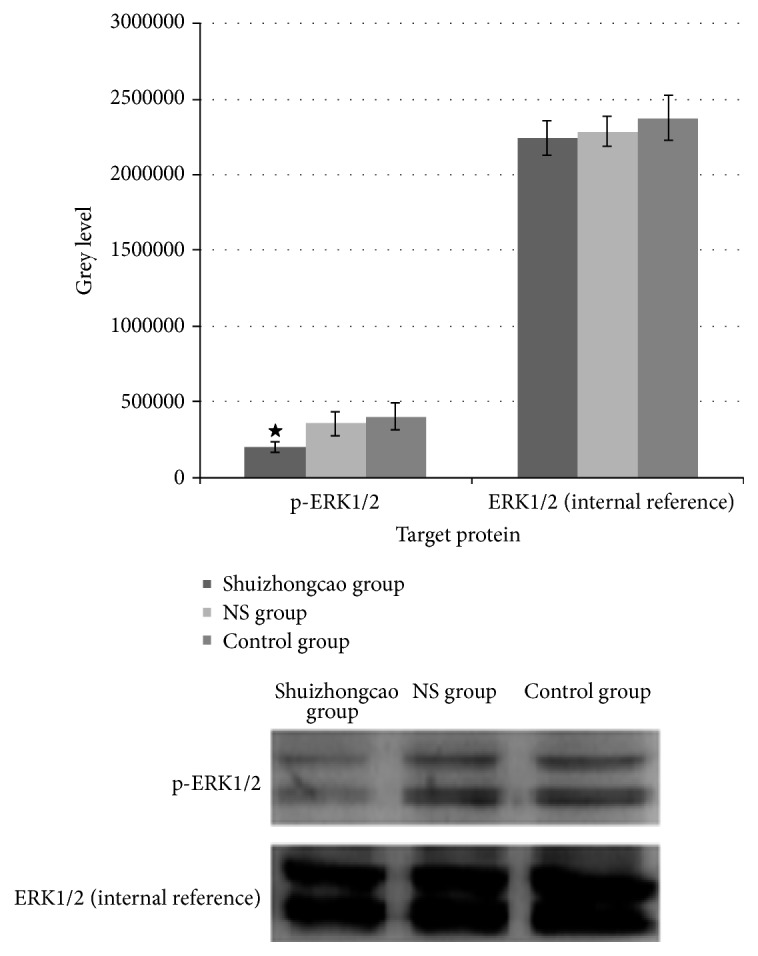
Expression of phosphorylated ERK1/2.

The results demonstrated that Shuizhongcao-containing serum led to suppressed expression of phosphorylated ERK1/2 and thus inhibited ERK mediated proliferation pathway. The difference of phosphorylated ERK1/2 expression level between Shuizhongcao group and control group, as well as NS group and control group, was statistically significant (*P* < 0.05). See Figure 3.

#### 4.4.3. Expression of Phosphorylated AKT

**Figure 4:**
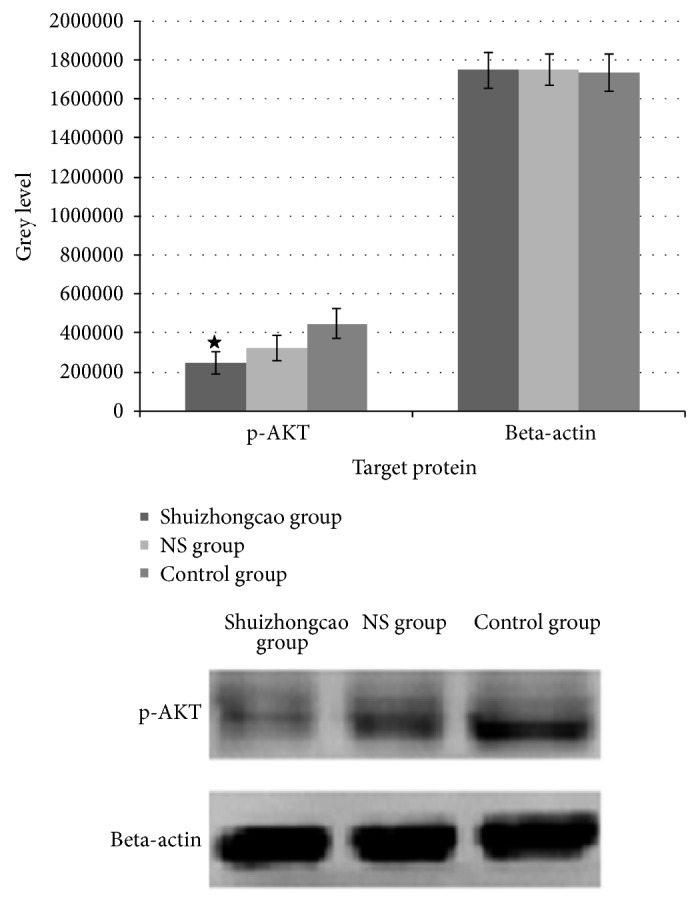
Expression of phosphorylated AKT.

The results demonstrated that Shuizhongcao-containing serum suppressed the expression of phosphorylated AKT. Differences between the phosphorylated AKT expression level of Shuizhongcao group and that of two other groups were statistically significant (*P* < 0.05), respectively. The difference between NS group and control group was not significant. See Figure 4.

### 4.5. PCR Results of IL-10, IL-12a, and IL-12b

Considering the large variations in PCR measurements, a normal group was set up, that is, using drug-containing rat serum without any treatment as reference. The measurements in the control group and Chinese medicine group were, respectively, divided by the values in the normal group to calculate relative expression amounts to more objectively reflect the expression difference.

**Figure 5:**
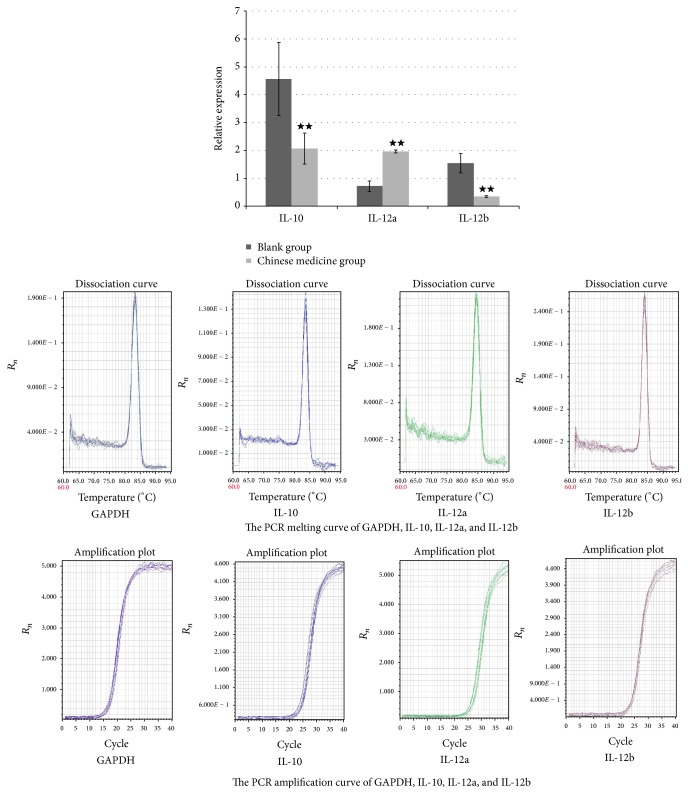
Relative expression of IL-10, IL-12a, and IL-12. Note: ★★ compared with control group, *P* < 0.01.

The results showed that levels of IL-10 expression in both Chinese medicine group and control group were significantly increased (*P* < 0.01), compared with normal control group. The expression levels of IL-10 and IL-12a in Chinese medicine group were significantly decreased compared with those in control group (*P* < 0.01). When compared with normal group, IL-12a expression level was increased in Chinese medicine group while it decreased in control group, with significant differences (*P* < 0.01). When compared with normal group, the IL-12b expression level was decreased in Chinese medicine group while it increased in control group, with significant differences. See Figure 5.

## 5. Discussion

Fibroblast proliferation is crucial for the healing of oral ulcers. CCK8 assay indicated that Shuizhongcao Granule did enhance fibroblast proliferation. Western blot showed that the serum containing Shuizhongcao Granule inhibited AKT expression in fibroblasts. We supposed that fibroblast proliferation was related to inflammation inhibition caused by AKT inhibition. However, this needs to be confirmed by controlled trial of gene silencing. We aimed to identify the effect of Shuizhongcao Granule on oral ulcers in animal and cell models using CCK8 assay, and the results confirmed that fibroblast proliferation was enhanced. Subsequent Western blot and PCR were intended to find out the reasons for this and to collect data for analysis.

According to our preliminary research, Shuizhongcao Granule was able to increase the red blood cell Cab receptor rosette formation rate but decrease immune complex rosette formation rate in red blood cells [9]. We also demonstrated that, in ROU, Shuizhongcao Granule was capable of increasing the percentage of CD3 and CD4 positive cells, CD4/CD8 ratio, and serum level of IL-12 and decreasing serum level of IL-10 [10]. Through the rat ROU model, we compared the recovery time of ulcers in Shuizhongcao group and model group and demonstrated the efficacy of Shuizhongcao Granule in treating ROU. The* in vivo* effect of inflammation inhibition of Shuizhongcao was demonstrated by levels of IL-4, IL-8, and GM-CSF in rat serum.

Based on the preliminary research, this study focused on exploring the pathways associated with inflammation. NF-*κ*B is a canonical pathway associated, among others, with inflammation. It has also been reported that NF-*κ*B regulates several hundred genes involved in cell growth, differentiation, and apoptosis [11]. Here we reported that the level of phosphorylated P65 in Chinese medicine group was significantly decreased compared with two other groups. Since the expression of NF-*κ*B pathway mainly relies on the binding of phosphorylated P65 on the particular gene target in the nucleus, NF-*κ*B pathway might be inhibited in the Chinese medicine group. However, further experiments showed that, after the treatment of Chinese medicine, expression levels of phosphorylated I*κ*B*α* and phosphorylated IKK*αβ* increased, while IKK*β* expression was decreased, which was contrary to what should be presented by the canonical pathway. Therefore, we speculate that Shuizhongcao Granule suppresses the activation of NF-*κ*B pathway through inhibiting an alternative unknown pathway, rather than inhibiting the canonical pathway.

Our preliminary data showed that serum containing Shuizhongcao facilitated fibroblast proliferation (or inhibited inflammation lesions). Thus we speculated that serum containing Shuizhongcao Granule would be able to activate ERK pathway, promote cell proliferation, and suppress apoptosis. However, the results demonstrated that drug-containing serum inhibited the expression of phosphorylated ERK1/2, which are the key players of ERK pathway. Therefore, we inferred that the ability of Shuizhongcao Granule to promote cell proliferation is through the inhibition of p-AKT expression and thus to regulate the NF-*κ*B pathway.

As a major player on antiapoptotic pathway, AKT can exert a dominant negative effect and inhibit insulin-like growth factor 1 (IGF1) mediated cell growth; thus persistent activation of AKT inhibits PTEN mediated apoptosis. AKT also affects cell survival by an indirect effect on Pi3k-AKT and P53 [12]. It has been shown that [13–15] AKT indirectly affects NF-*κ*B and P53 and thus influences cell survival. By phosphorylating and activating kB kinase (IKK), AKT leads to the degradation of I*κ*B, an inhibitor of NF-*κ*B, and thus results in cytoplasm release and nuclear translocation of NF-*κ*B, which in turn activates its target genes and promotes cell survival. Our experiments showed that Chinese medicine containing serum significantly inhibited the level of phosphorylated AKT. Therefore, we inferred that it was the AKT-NF-*κ*B signaling pathway that Shuizhongcao Granule acted through on fibroblasts.

Our study confirmed the ability of Shuizhongcao to accelerate the recovery of ROU rat and further demonstrated that the mode of action was associated with inflammatory reaction inhibition. The mechanism underlying facilitation of fibroblast proliferation by Shuizhongcao is yet to be discovered. Our research provides an experimental rationale for the application of Shuizhongcao in clinical therapy.

## Figures and Tables

**Table 1:** Real-time PCR primers.

Gene	Primer sequence
GAPDH	GGAGCGAGATCCCTCCAAAAT
	GGCTGTTGTCATACTTCTCATGG

IL-10	GCTATGTTGCCTGCTCTTACTG
	TCTGGCTGACTGGGAAGTG

IL-12a	AGACATCACACGGGACAAAAC
	CACAGGGTCATCATCAAAGAAG

IL-12b	AGCACTCCCCATTCCTACTTCT
	AACGCACCTTTCTGGTTAC

**Table 2:** OD values of rat fibroblast following intervention by Chinese medicine containing serum and control serum ($\bar{X}\pm S$, *n* = 6).

Group	Time				Zero pores
	12 h	24 h	36 h	48 h	
5% control group	0.4944 ± 0.01294	0.6412 ± 0.03167	0.8769 ± 0.03631	1.0179 ± 0.04123	0.0834 ± 0.01123
10% control group	0.4240 ± 0.00884	0.5231 ± 0.02622	0.6874 ± 0.02147	0.7213 ± 0.01843	0.0782 ± 0.00873
15% control group	0.3713 ± 0.00968	0.4035 ± 0.01732	0.5589 ± 0.01483	0.6633 ± 0.01627	0.0761 ± 0.01374
5% Chinese medicine group	0.5833 ± 0.02643^★^	0.6934 ± 0.01216^★^	0.9088 ± 0.01736	1.0235 ± 0.03513	0.0851 ± 0.01165
10% Chinese medicine group	0.4780 ± 0.01637^★^	0.5687 ± 0.01072^★^	0.7189 ± 0.02987^★^	0.8636 ± 0.01532^★^	0.0801 ± 0.02198
15% Chinese medicine group	0.4083 ± 0.00996	0.4488 ± 0.00832^★^	0.5986 ± 0.01653^★^	0.7399 ± 0.01843^★^	0.0719 ± 0.01682
Blank group	0.5901 ± 0.02398	0.7287 ± 0.03128	0.9432 ± 0.03612	1.0213 ± 0.02871	0.0912 ± 0.01572

Note: ^★^ *P* < 0.05 between Chinese medicine group and control group at the same time point.

## References

[1] Tarakji B., Baroudi K., Kharma Y. (2012). The effect of dietary habits on the development of the recurrent aphthous stomatitis. *Nigerian Medical Journal*.

[2] Meng W., Dong Y., Liu J. (2009). A clinical evaluation of amlexanox oral adhesive pellicles in the treatment of recurrent aphthous stomatitis and comparison with amlexanox oral tablets: a randomized, placebo controlled, blinded, multicenter clinical trial. *Trials*.

[3] Tsuyuki S., Kawaguchi K., Kawata Y. (2012). Usefulness of antimycotic agents (itraconazole) in chemotherapy-induced mucositis of breast cancer patients. *Gan To Kagaku Ryoho*.

[4] Tappuni A. R., Kovacevic T., Shirlaw P. J. (2013). Clinical assessment of disease severity in recurrent aphthous stomatitis. *Journal of Oral Pathology & Medicine*.

[5] Babaee N., Moslemi D., Khalilpour M. (2013). Antioxidant capacity of calendula officinalis flowers extract and prevention of radiation induced oropharyngeal mucositis in patients with head and neck cancers: a randomized controlled clinical study. *Daru*.

[6] Caputo B. V., Filho G. A. N., dos Santos C. C., Okida Y., Giovani E. M. (2012). Laser therapy of recurrent aphthous ulcer in patient with HIV infection. *Case Reports in Medicine*.

[7] Yang X. M., Wang X. H., Chen L. F. (2012). Effects of dihydromyricetin on tumor necrosis factor and NF-kappaB p65 of RAU rats. *Zhongguo Zhong Yao Za Zhi*.

[8] Jin T., Shen M.-H., Sun Y.-F. (2009). Shuizhongcao decoction treatment chemotherapy induced oral ulcer. *Chinese Archives of Traditional Chinese Medicine*.

[9] Shen M. H., Ruan S. M., Bao M. H. (2009). Effect of shuizhongcao granule on cellular immune function of experimental animal with recurrent aphthous stomatitis. *Zhongguo Zhong Xi Yi Jie He Za Zhi*.

[10] Shen M. H., Ruan S. M., Bao M. H. (2008). Research of Shuizhongcao Granule on experimental animal recurrent oralulcer of the red cell immune function. *Chinese Archives of Traditional Chinese Medicine*.

[11] Siomek A. (2012). NF-*κ*B signaling pathway and free radical impact. *Acta Biochimica Polonica*.

[12] Jeong S.-J., Pise-Masison C. A., Radonovich M. F., Park H. U., Brady J. N. (2005). Activated AKT regulates NF-*κ*B activation, p53 inhibition and cell survival in HTLV-1-transformed cells. *Oncogene*.

[13] Busch F., Mobasheri A., Shayan P., Stahlmann R., Shakibaei M. (2012). Sirt-1 is required for the inhibition of apoptosis and inflammatory responses in human tenocytes. *The Journal of Biological Chemistry*.

[14] Busch F., Mobasheri A., Shayan P. (2012). Resveratrol modulates interleukin-1beta-induced phosphatidylinositol 3-kinase and nuclear factor kappaB signaling pathways in human tenocytes. *The Journal of Biological Chemistry*.

[15] Park Y. D., Kim Y. S., Jung Y. M. (2012). Porphyromonas gingivalis lipopolysaccharide regulates interleukin (IL)-17 and IL-23 expression via SIRT1 modulation in human periodontal ligament cells. *Cytokine*.

